# Localized Surface Plasmon Resonance-Based Gas Sensor with a Metal–Organic-Framework-Modified Gold Nano-Urchin Substrate for Volatile Organic Compounds Visualization

**DOI:** 10.3390/s25216522

**Published:** 2025-10-23

**Authors:** Cong Wang, Hao Guo, Bin Chen, Jia Yan, Fumihiro Sassa, Kenshi Hayashi

**Affiliations:** 1 Graduate School of Information Science and Electrical Engineering, Kyushu University, Fukuoka 819-0395, Japan; cong.929@s.kyushu-u.ac.jp (C.W.);; 2 Chongqing Key Laboratory of Non-Linear Circuit and Intelligent Information Processing, College of Electronic and Information Engineering, Southwest University, Chongqing 400715, China; 3 College of Artificial Intelligence, Southwest University, Chongqing 400715, China

**Keywords:** localized surface plasmon resonance, Au nano-urchins, ZIF-8, volatile organic compounds, gas visualization

## Abstract

Volatile organic compound (VOC) monitoring is crucial for environmental safety and health, but conventional gas sensors often suffer from poor selectivity or lack spatial information. Here, we report a localized surface plasmon resonance (LSPR) gas sensor based on Au nano-urchins coated with a zeolitic imidazolate framework (ZIF-8) for both the quantitative detection and visualization of VOCs. Substrates were fabricated by immobilizing Au nano-urchins (~90 nm) on 3-aminopropyltriethoxysilane-modified glass and subsequently growing ZIF-8 crystals (~250 nm) for different durations. Scanning electron microscopy and optical analysis revealed that 90 min of ZIF-8 growth provided the optimal coverage and strongest plasmonic response. Using a spectrometer-based LSPR system, the optimized substrate exhibited clear, concentration-dependent responses to three representative VOCs, 2-pentanone, acetic acid, and ethyl acetate, over nine concentrations, with detection limits of 12.7, 14.5, and 36.3 ppm, respectively. Furthermore, a camera-based LSPR visualization platform enabled real-time imaging of gas plumes and evaporation-driven diffusion, with differential pseudo-color mapping providing intuitive spatial distributions and concentration dependence. These results demonstrate that ZIF-8-modified Au nano-urchin substrates enable sensitive and reproducible VOC detection and, importantly, transform plasmonic sensing into a visual modality, offering new opportunities for integrated LSPR–surface-enhanced Raman scattering dual-mode gas sensing in the future.

## 1. Introduction

Volatile organic compounds (VOCs) are ubiquitous in both indoor and outdoor environments, and they are closely associated with air quality, industrial safety, and human health [1]. Accurate VOC monitoring is, therefore, essential for environmental protection [2,3,4,5], occupational safety [6], and medical diagnostics [7,8,9,10]. However, existing VOC sensors often face trade-offs between sensitivity, selectivity, and operational requirements, and many conventional designs provide only point detection without spatial information, limiting their applicability in scenarios that demand real-time visualization of gas distributions.

Recent advances in VOC sensing span multiple transduction families, including mass-sensitive quartz crystal microbalance (QCM), chemiresistive metal-oxide semiconductors (MOS), field-effect-transistor (FET) devices, and non-plasmonic optical methods (e.g., NDIR, photoacoustic, colorimetric/fluorometric, and interferometric sensing). These approaches have collectively pushed the limits of detection from the ppm down to the ppb or even ppt regime in laboratory settings while facing trade-offs among selectivity, power, and form factor. As a representative mass sensor, QCM translates analyte adsorption on a functional coating into resonance-frequency shifts and, when arrayed with diverse sorptive layers, enables pattern-based VOC recognition [9,11,12,13,14,15,16]. However, QCMs are limited to point measurements, require complex frequency control electronics, and are difficult to miniaturize into imaging systems. Chemiresistive MOS sensors rely on changes in electrical resistance due to the redox reaction between gas molecules and adsorbed oxygen species on metal oxide surfaces, which are widely used because of their simple structure, high sensitivity, and low cost [17,18,19,20]. Nevertheless, most MOS sensors must operate at high temperatures to activate surface reactions, resulting in high power consumption, poor selectivity, and short lifetime. FET-based gas sensors detect the modulation of charge carriers in a semiconductor channel when target molecules interact with the sensing layer. These devices offer room-temperature operation, high signal amplification, and facile integration with electronic circuits [6,21,22,23,24]. Despite their ppb-level sensitivity, FET sensors are often affected by humidity and fabrication variability, and they provide only local electrical readouts rather than spatially continuous information. Non-plasmonic optical routes identify VOCs through their interaction with light—via infrared absorption, fluorescence, or chromogenic reactions. Techniques such as non-dispersive infrared, photoacoustic, and Fourier-transform infrared spectroscopy offer high molecular specificity and quantitative accuracy [25,26,27], while colorimetric and fluorescence sensor arrays provide intuitive visual responses to chemical vapors [28,29,30].

Localized surface plasmon resonance (LSPR) has emerged as a promising optical sensing strategy owing to its rapid response, label-free operation, and compatibility with room-temperature measurements. The sensing principle relies on monitoring the shifts in the plasmon resonance caused by refractive index variations near metallic nanostructures [31,32]. LSPR substrates with tailored nanostructures have achieved part-per-million to sub-part-per-million-level gas detection sensitivity [33]. Among the different nanostructures, gold nano-urchins are particularly attractive because their spiky morphology generates strong localized electromagnetic “hot spots” and enhances plasmonic coupling, leading to pronounced sensitivity and changes in the surrounding refractive index [34,35]. Moreover, gold provides superior chemical stability and oxidation resistance compared to silver, making it suitable for long-term gas-phase operation.

Metal–organic frameworks (MOFs), such as zeolitic imidazolate framework-8 (ZIF-8), have also been increasingly integrated with plasmonic substrates to improve the gas adsorption and sensing performance. ZIF-8 has a high surface area and well-defined micropores, which can enrich analyte molecules in the vicinity of plasmonic nanostructures, thereby amplifying the LSPR response [36,37]. Combining Au nano-urchins with ZIF-8, therefore, provides a synergistic strategy: the Au nano-urchins contribute strong LSPR sensitivity, while ZIF-8 enhances the molecular adsorption close to the sensing surface. This synergy boosts both sensitivity (via local refractive-index modulation) and image contrast under broadband illumination, making LSPR imaging a practical route for real-time VOC plume visualization.

The visualization of gas plumes has become an emerging development in sensor science. While surface-enhanced Raman scattering (SERS) arrays can deliver chemical-specific imaging, they generally require point-by-point spectral acquisition, which is time-consuming [38]. Fluorescent odor imaging and colorimetric arrays can generate intuitive outputs, but they often lack universality or require analyte-specific chemistries [39]. In contrast, LSPR imaging enables wide-field monitoring of the refractive index changes across the substrate surface under simple broadband illumination and camera detection [32,33]. This approach effectively transforms chemical sensing into a visual modality, offering intuitive and dynamic insights into gas dispersion.

In this study, we developed ZIF-8-modified Au nano-urchin substrates for both quantitative VOC detection and real-time visualization. By tuning the ZIF-8 growth time, we identified the optimal substrate configuration. The sensing performance was first characterized using a spectrometer-based LSPR system, demonstrating concentration-dependent responses toward three representative VOCs: 2-pentanone, acetic acid, and ethyl acetate. Furthermore, a camera-based LSPR imaging system was developed to directly visualize the gas distribution generated by controlled flow or droplet evaporation. This work highlights how combining Au nano-urchins and ZIF-8 can enhance plasmonic sensing and enable intuitive visualization, paving the way for future integrated LSPR–SERS multimodal gas sensing platforms [40].

## 2. Materials and Methods

### 2.1. Synthesis of the Au Nano-Urchins

The Au nano-urchins were synthesized by a seed-mediated growth method developed from previously reported protocols [35,41]. First, spherical gold seeds were prepared by citrate reduction. In brief, 75 µL of 100 mM HAuCl_4_ solution was added to 30 mL of vigorously stirred and boiling deionized water, and then 900 µL of 1 *w*/*v*% trisodium citrate solution was introduced. The reaction mixture was maintained under boiling conditions until a characteristic red-wine color appeared, indicating the successful nucleation of Au seeds. The resulting seed solution was cooled to room temperature under continuous stirring. For the growth of the Au nano-urchins, 25 µL of 100 mM HAuCl_4_ solution was mixed with 9.6 mL of deionized water under vigorous stirring. To this mixture, 150 µL of the as-prepared seed solution, 22 µL of 1 *w*/*v*% trisodium citrate solution, and 1.2 mL of 30 mM hydroquinone solution were sequentially added. The suspension was stirred for an additional 30 min, during which branched Au nanoparticles with a spiky urchin-like morphology formed.

### 2.2. Fabrication of the ZIF-8-Modified Au Nano-Urchin Substrates

**Figure 1:**
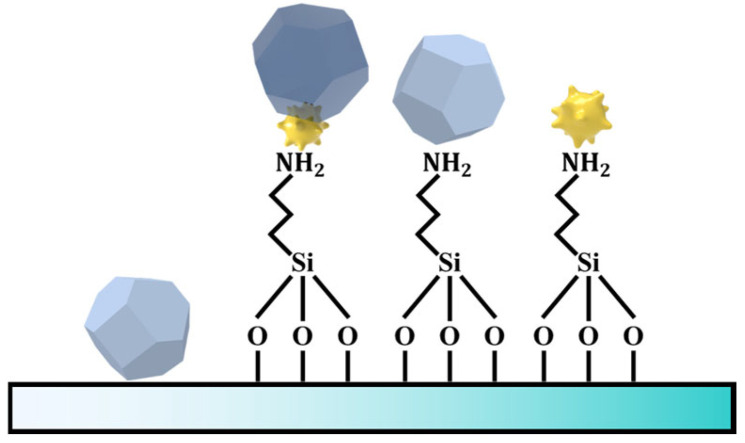
Schematic illustration of the substrate fabrication process. The glass surface was first functionalized with 3-aminopropyltriethoxysilane, forming—NH_2_ groups on the substrate. These amino groups enabled the electrostatic adsorption of nanoparticles (e.g., ZIF-8 crystals and Au nano-urchins), resulting in the formation of a functionalized plasmonic sensing surface.

Glass slides were sequentially ultrasonicated in acetone, ethanol, and deionized water, dried under nitrogen, and then silanized by immersion in 3-aminopropyltriethoxysilane (APTES)/ethanol solution (1:100, *v/v*) for 1 h at room temperature [34]. The substrates were ultrasonically rinsed three times with ethanol (5 min each time), dried with nitrogen, and cured in a muffle furnace at 120 °C for 3 h. The APTES-functionalized glass slides were then incubated in the as-synthesized Au nano-urchin colloid overnight to allow dense nanoparticle adsorption, followed by gentle rinsing with deionized water and drying with nitrogen. For ZIF-8 growth, 1.5 mL of 25 mM Zn(NO_3_)_2_ solution was mixed with 1.5 mL of 50 mM 2-methylimidazole solution, and the Au-nano-urchin-coated substrates were immediately immersed in the precursor mixture. After deposition, the substrates were removed, rinsed with ethanol to remove residual precursors, and dried under nitrogen. The stepwise fabrication process, including silanization of the glass slide with APTES, immobilization of the Au nano-urchins, and subsequent ZIF-8 growth, is schematically illustrated in Figure 1.

### 2.3. Gas Generation and Optical Measurement Systems

**Figure 2:**
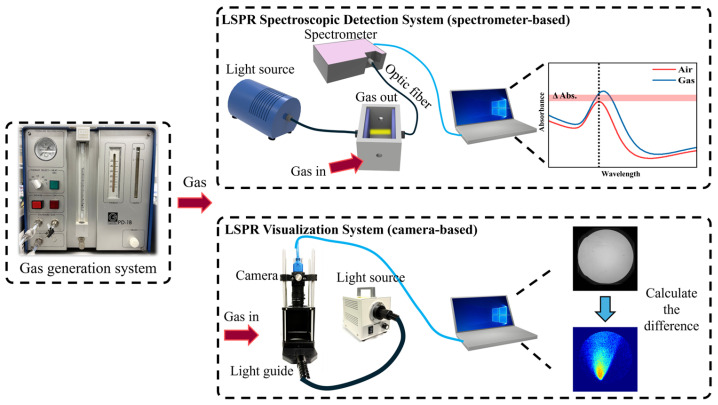
Schematic of the experimental platforms: gas generation system (**left**), localized surface plasmon resonance (LSPR) spectroscopic detection system (**top right**), and LSPR visualization system (**bottom right**). The spectrometer-based LSPR system measures transmittance spectra through the gas chamber, and the dashed line in the inset indicates the wavelength position used for calculating the absorbance change (ΔA=T_air−T_gas). The camera-based system captures transmission images to visualize spatial gas distributions by differential image processing, where the pseudo-color mapping (cv2.COLORMAP_JET) represents the magnitude of transmittance change—red indicates stronger changes and blue indicates weaker changes.

A dedicated gas generation system was used to generate controlled concentrations of the target VOCs, as shown in Figure 2. The desired concentration was obtained by selecting tubing with an appropriate inner diameter for the liquid reservoir and by tuning the total carrier-gas flow rate via flow meters. The concentration of the vapor-phase analyte introduced into the detection chamber was estimated based on the mass loss of the liquid analyte during controlled evaporation. The vapor concentrations were calculated by the following [35]:

(1) $$C_{ppm}=\frac{K\;\times \;D_{r}\;\times \;1000}{F},$$

where $C_{ppm}$ is the standard vapor concentration (ppm), $D_{r}$ is the diffusion rate (μg/min), and *F* is the flow rate of the dilute air (L/min). The diffusion rate $D_{r}$ was experimentally obtained by measuring the mass loss of the liquid analyte reservoir before and after each run and dividing it by the elapsed time, representing the evaporation rate of the analyte under the given flow conditions. *K* (dimensionless) is the factor used to convert the vapor weight to the volume [35]:

(2) $$K=\frac{22.4\;\times \;\left(273\;+\;t\right)\;\times \;760}{M\;\times \;273\;\times \;P},$$

where *M* is the molecular weight, *t* is the temperature in the gas chamber (°C), and *P* is the gas pressure (760 mmHg). The mixed gas was delivered to the downstream measurement platforms through polytetrafluoroethylene (PTFE) tubing.

The LSPR spectroscopic detection system (spectrometer-based, top right part of Figure 2) consisted of a light source, a custom PTFE gas chamber, two optical fibers, a fiber-optic spectrometer, and a computer. The two fibers were aligned in a transmission geometry across the LSPR sensor: one was coupled with the light source to illuminate the substrate at normal incidence, and the other collected the transmitted light and guided it into the spectrometer. The transmittance spectra were recorded and processed with OPwave+ software (version 4.17). The gas response was evaluated by calculating the absorbance change (Δ*A*):

(3) $$\Delta A=T_{air}-T_{gas},$$

where $T_{air}$ is the transmittance in air and $T_{gas}$ is the transmittance under the target gas.

The LSPR visualization system (camera-based, bottom right part of Figure 2) comprised a light source, a light guide for the surface-emitting type, optical mounts, a camera, and a computer. The LSPR substrate was placed above the light guide while gas was introduced across its surface. Image sequences were captured at a fixed exposure, and the subsequent analysis included frame-to-frame differential imaging (current frame minus baseline) followed by pseudo-color mapping. This processing amplified the subtle transmittance changes to visually discernible spatial gas distributions, enabling rapid, wide-field visualization of gas plumes across the sensor.

### 2.4. Experimental Setup for Gas Visualization

**Figure 3:**
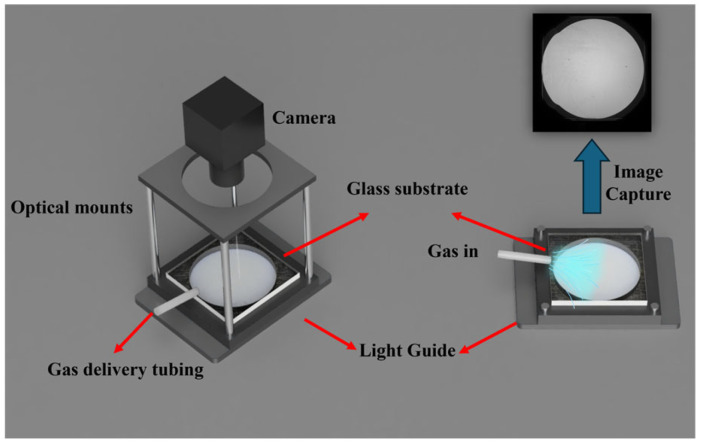
Schematic of the flow-based gas introduction experiment using the camera-based LSPR system.

**Figure 4:**
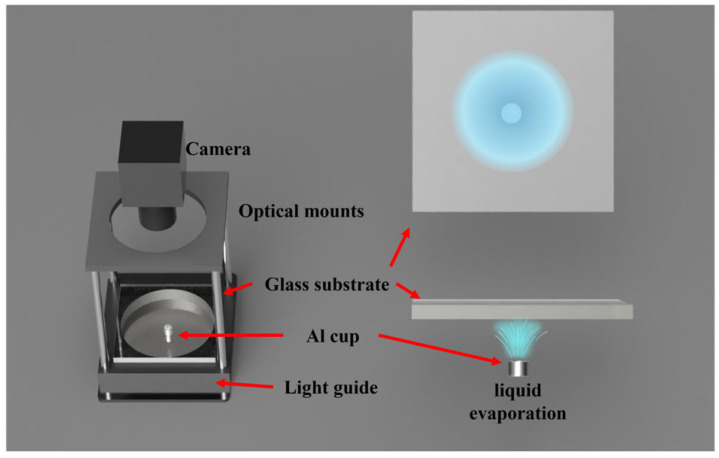
Schematic of the droplet evaporation experiment for volatile organic compound (VOC) visualization.

Two experimental configurations were used to visualize the gas distributions using the camera-based LSPR system, as shown in Figure 3 and Figure 4. For both setups, a light guide was positioned beneath the glass substrate to generate uniform illumination, and a camera mounted on top captured transmission images for subsequent analysis.

In the first experimental configuration (Figure 3), the target vapors at controlled concentrations were generated by a gas delivery system and introduced on the substrate surface through a delivery tube. Each measurement consisted of three phases: 60 s of air purging, 120 s of target-gas exposure, and 120 s of air recovery. The transmitted light through the substrate was continuously recorded from the top view to extract the spatial distribution of the gas.

In the second experimental configuration (Figure 4), the gas source was produced by natural evaporation of a liquid droplet. A small aluminum cup (5 mm diameter) was placed between the substrate and the light guide. The glass substrate was mounted facing downward above the cup, into which 30 μL of liquid was dispensed. The liquid gradually evaporated into the surrounding air. Transmission images were continuously captured for 36 min (3600 frames in total).

To ensure quantitative and reproducible imaging, the camera parameters were fixed throughout all experiments. The FLIR Blackfly S (BFS-U3-23S3M-C, Teledyne FLIR Integrated Imaging Solutions Inc., Richmond, BC, Canada, mono, 12-bit ADC) equipped with a TECHSPEC^®^ UC series fixed-focus lens (33-301, Edmund Optics Inc., Barrington, NJ, USA) was used to capture transmission images. The lens focus was adjusted to precisely align with the substrate plane, and all automatic camera functions—including auto-exposure, gain, and white-balance—were disabled to maintain consistent acquisition conditions. The LED light source (HAYASHI-REPIC Lumina Ace LA-HDF100NA, 6500 K, Hayashi Repic Co., Ltd., Tokyo, Japan) together with a surface-emitting light guide generated a uniform 60 mm × 60 mm illumination field. The light intensity was tuned so that the 12-bit analog-to-digital converter operated below its saturation range. Image sequences were stored in mono-16 TIFF format to preserve the full dynamic range.

Each captured frame was processed through a multi-stage image-analysis pipeline to enhance the minute transmittance variations caused by local refractive-index changes. First, the effective sensing region corresponding to the optical transmission area of the mounts was isolated by applying a circular binary mask, thereby defining the region of interest (ROI) for subsequent analysis. The baseline image was obtained by averaging the first or last five frames, depending on the experiment type, and each subsequent frame was subtracted from this baseline to produce a differential image:

(4) $$I_{diff}\left(x,y,t\right)=I\left(x,y,t\right)-I_{baseline}\left(x,y\right),$$

This differential operation effectively eliminated static background nonuniformity. A Gaussian filter (kernel size 7 × 7, σ = 2) was then applied to suppress high-frequency noise, followed by thresholding to remove residual low-level fluctuations. The resulting intensity values were normalized to an 8-bit scale to maintain visual consistency across frames. A non-local means (NLM) filter was subsequently used for further denoising, and the processed matrices were converted into pseudo-color maps using the JET colormap. The color scale was linearly normalized to 0–255, where warmer colors correspond to larger positive transmittance changes (ΔA), indicating stronger local LSPR responses. Each frame was finally exported as a PNG file for visualization and video generation.

In this imaging configuration, each camera pixel corresponds to an optically averaged region on the LSPR substrate rather than a single nanoparticle. The illuminated area of 60 mm × 60 mm was projected onto 1200 × 1200 pixels, yielding an effective spatial resolution of approximately 50 µm per pixel. Each pixel thus represents the mean transmittance of a local area containing numerous Au nano-urchins, providing a mesoscale mapping of refractive-index variations across the substrate. The surface-emitting light guide produced a uniform, quasi-diffuse illumination, under which each pixel primarily collects light transmitted through its corresponding local area. Because the illumination and detection are both aligned along the substrate normal and the optical paths associated with different pixels are spatially distinct, the influence of lateral scattering between neighboring regions is minimal. Consequently, crosstalk between adjacent pixel signals is negligible, and the pixel-wise intensity change can be regarded as an independent indicator of the local LSPR response on the substrate.

## 3. Results and Discussion

### 3.1. Morphological Evolution and Optical Properties of the ZIF-8-Modified Au Nano-Urchin Substrates

**Figure 5:**
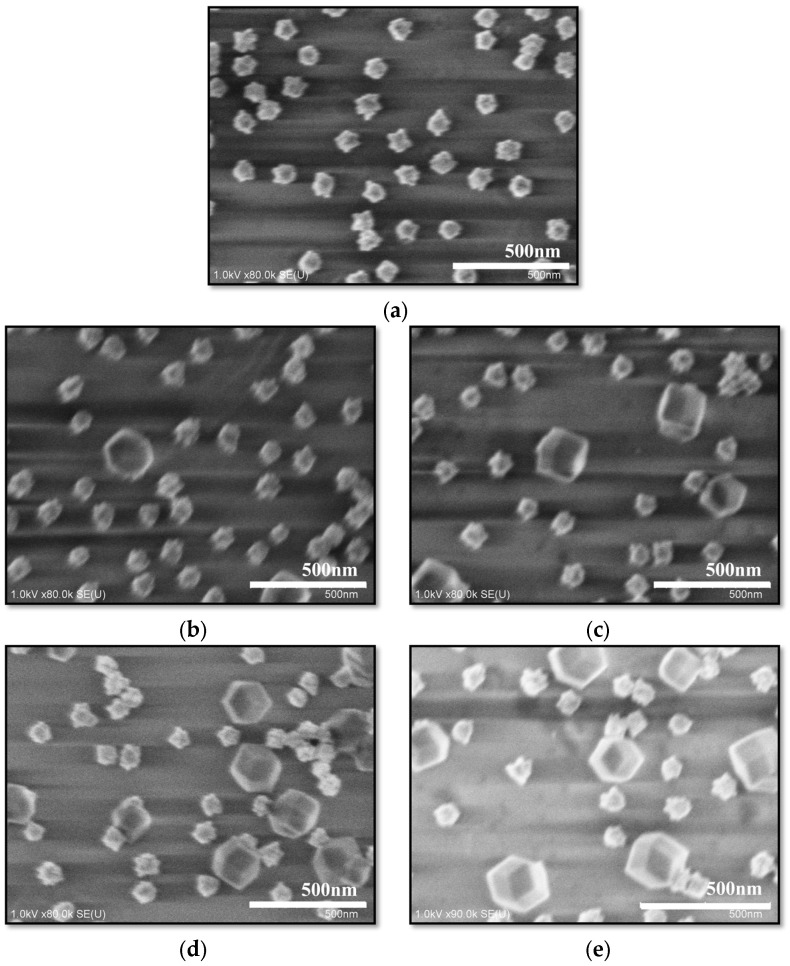
Scanning electron microscopy images of the Au nano-urchin substrates produced with different ZIF-8 growth times: (**a**) 0 min, Au nano-urchins with ~90 nm diameter and sharp tips; (**b**) 30 min, initial appearance of ~200 nm rhombic dodecahedral ZIF-8 crystals; (**c**) 60 min; (**d**) 90 min; and (**e**) 120 min. The ZIF-8 crystal density increased with the growth time and reached saturation at ~90 min ZIF-8 growth time.

The surface morphologies of the Au nano-urchin substrates prepared with different ZIF-8 growth times were examined by scanning electron microscopy (SEM, SU8000, Hitachi High-Technologies Corp., Tokyo, Japan) (Figure 5a–e). Before ZIF-8 deposition, the Au nano-urchins exhibited an average diameter of ~90 nm with sharp protrusions and appropriate interparticle spacing (Figure 5a), features that are favorable for strong plasmonic coupling. After 30 min of ZIF-8 growth, ZIF-8 crystals with rhombic dodecahedral morphology (~200 nm in diameter) appeared on the substrate (Figure 5b). With increasing immersion time, the density of ZIF-8 crystals progressively increased (Figure 5c,d). For 120 min ZIF-8 growth (Figure 5e), the particle density was comparable to that observed for 90 min ZIF-8 growth, suggesting that the ZIF-8 growth had reached saturation. Additional large-area SEM images are provided in the Supplementary Information (Figure S1), showing that the ZIF-8 crystals have uniform particle sizes and that only the deposition density varies with growth time. These results confirm the consistent quality and uniformity of the ZIF-8 layer obtained at 90 min growth.

**Figure 6:**
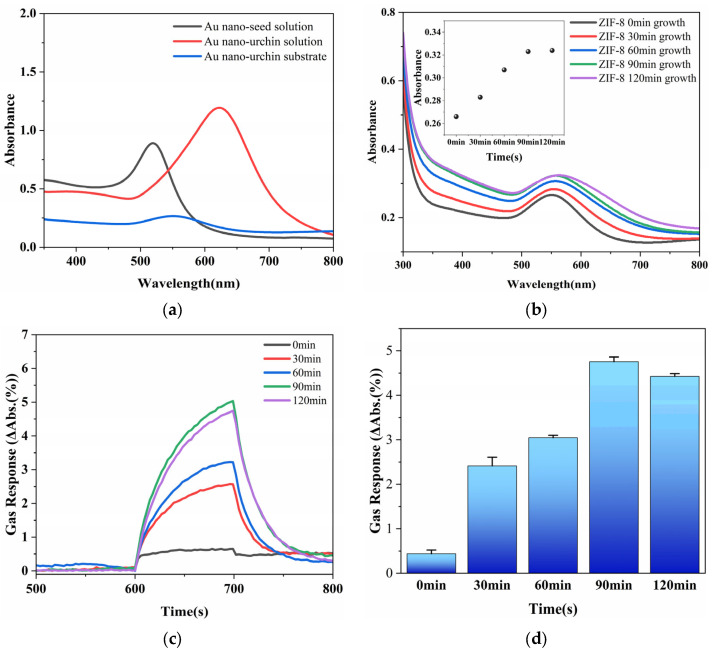
Optical and gas sensing characterization of the ZIF-8-modified Au nano-urchin substrates. (**a**) Ultraviolet–visible absorption spectra of the Au seed solution (plasmon peak at 519 nm), Au nano-urchin colloid (red-shifted to 622 nm), and Au nano-urchins deposited on glass (551 nm). (**b**) Absorption spectra of the substrates after different ZIF-8 growth times (0, 30, 60, 90, and 120 min), showing a progressive red-shift and increased absorbance up to 90 min, followed by saturation (the insert shows the peak absorbance versus the growth time). (**c**) Real-time gas responses at 558 nm for the five substrates exposed to 381 ppm 2-pentanone (600 s air baseline, gas exposure for 100 s, and then air purging). (**d**) Average responses during the final 20 s of gas exposure, indicating that the 90 min ZIF-8-coated substrate exhibited the maximum response.

The corresponding optical absorption spectra of the Au nano-urchin substrates are shown in Figure 6a,b. For comparison, the optical absorption spectrum of the Au seed solution is also shown in Figure 6a, which exhibits a characteristic plasmon peak at 519 nm. After secondary growth, the absorption maximum of the Au nano-urchin colloid red-shifted to 622 nm, which is consistent with the larger size and branched morphology of the Au nano-urchins. Once the Au nano-urchin film was deposited on the glass substrate, it showed an absorption peak at 551 nm. During ZIF-8 growth (Figure 6b), both the LSPR peak position and absorbance gradually increased for the first 90 min, indicating progressive MOF deposition and enhanced light–matter interactions. After 90 min, no significant spectral changes were observed, in agreement with the SEM observations.

To assess the sensing performance, 381 ppm 2-pentanone vapor was introduced using the gas generation system (600 s air baseline, 100 s gas exposure, and then purging with air). The real-time transmission responses at 558 nm for the substrates prepared with different ZIF-8 growth times (0, 30, 60, 90, and 120 min) are shown in Figure 6c. All of the samples exhibited a clear increase in the gas response upon gas exposure, with the response magnitude increasing from 0 to 90 min ZIF-8 growth and then slightly decreasing for 120 min ZIF-8 growth. The average responses during the final 20 s of exposure are summarized as a bar chart in Figure 6d, which highlights that the 90 min ZIF-8-coated substrate exhibited the strongest response. These results indicate that 90 min ZIF-8 growth provides the optimal balance between the MOF coverage and efficient analyte diffusion, and therefore, this substrate was selected for subsequent experiments.

**Figure 7:**
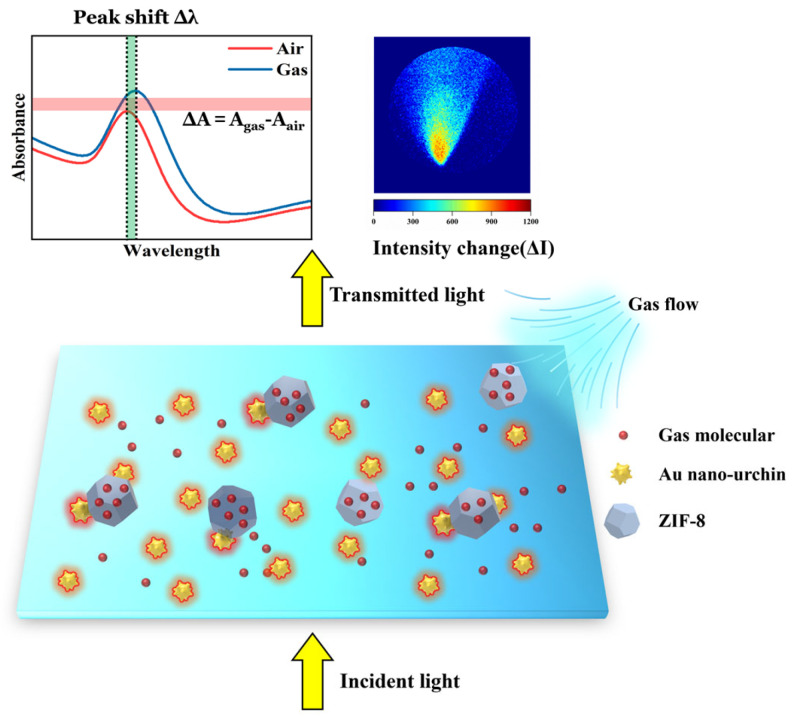
Schematic illustration of the synergistic sensing mechanism of the Au nano-urchin/ZIF-8 substrate. Gas molecules enriched within the porous ZIF-8 layer modify the local refractive index near the plasmonic “hot spots” generated by Au nano-urchins, thereby altering the localized surface plasmon resonance condition. This perturbation leads to measurable optical signal changes: (**left**) resonance peak shifts (∆λ) or absorbance variations (∆A) in the spectrometer-based LSPR system, and (**right**) pixel-wise intensity changes (ΔI) in the camera-based imaging system, which visualizes the spatial distribution of the gas plume. In the upper-left subfigure, the green-shaded area represents the spectral peak shift, the red-shaded area denotes the absorbance change, and the dashed line indicates the reference resonance peak position.

A schematic illustration of the sensing mechanism of the Au nano-urchin/ZIF-8 substrate is shown in Figure 7. The Au nano-urchins (yellow spiky particles) generate localized surface plasmon resonances at their sharp tips, forming electromagnetic “hot spots” that are highly sensitive to the refractive index of the surrounding medium. The ZIF-8 crystals (blue polyhedra), with a porous rhombic-dodecahedral structure, enrich gas molecules (red spheres) near these plasmonic regions. Upon gas exposure, the adsorbed molecules increase the local refractive index around the Au nano-urchins, perturbing the resonance coupling between incident light and the collective oscillation of conduction electrons. This perturbation manifests as measurable optical signal changes in two modes. In the spectrometer-based LSPR system, the transmitted spectra exhibit either a resonance peak shift (∆λ) or an absorbance variation (∆A) at a selected wavelength. In the camera-based LSPR imaging system, the same plasmonic modulation is captured as pixel-wise intensity differences (ΔI) in monochromatic transmission images, enabling spatially resolved visualization of the gas distribution. Thus, the local refractive-index modulation induced by molecular adsorption is directly converted into the observed spectral or intensity variations. The synergistic effect of plasmonic field enhancement from Au nano-urchins and molecular enrichment by ZIF-8 accounts for the amplified and reproducible gas responses observed experimentally.

### 3.2. Gas Sensing Performance of Different VOCs Using the Spectrometer-Based System

**Figure 8:**
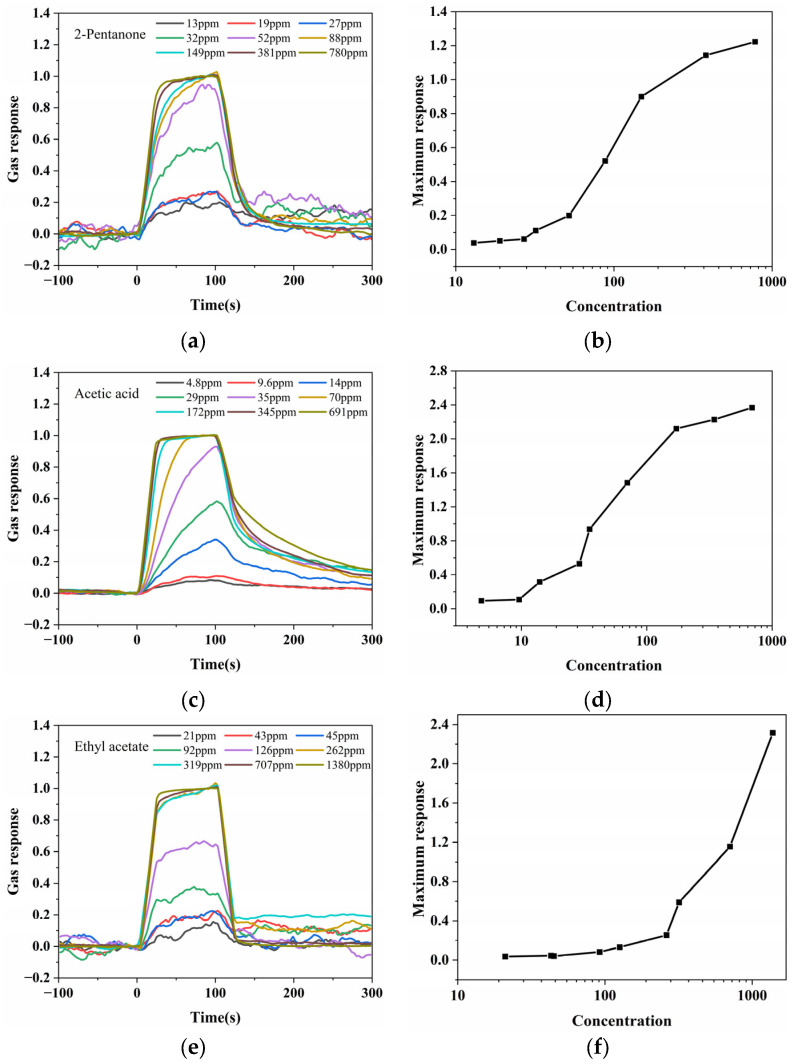
Gas sensing performance of the ZIF-8-modified Au nano-urchin substrate (90 min ZIF-8 growth) for three representative VOCs. Normalized dynamic response curves at 558 nm for nine concentrations of (**a**) 2-pentanone, (**c**) acetic acid, and (**e**) ethyl acetate (0 s corresponds to the onset of gas injection; weaker responses were compressed after normalization for clarity). Average response values during the final 20 s of gas exposure plotted against the analyte concentration (logarithmic *x* axis) for (**b**) 2-pentanone, (**d**) acetic acid, and (**f**) ethyl acetate, showing that the maximum response monotonically increased with increasing analyte concentration for all three VOCs.

The sensing performance of the ZIF-8-modified Au nano-urchin substrate (90 min ZIF-8 growth) was evaluated for three representative VOCs—2-pentanone, acetic acid, and ethyl acetate—using the LSPR spectroscopic detection system. The normalized responses at 558 nm for nine different concentrations of each analyte are summarized in Figure 8.

For 2-pentanone (Figure 8a), acetic acid (Figure 8c), and ethyl acetate (Figure 8e), the normalized dynamic response curves clearly showed that higher analyte concentrations led to faster response increases when the gas flow was switched on at 0 s. Despite differences in absolute intensity, normalization allowed a direct comparison of the response kinetics for all concentrations, while weaker raw responses were appropriately compressed for clarity.

The average response values during the final 20 s of gas exposure for 2-pentanone, acetic acid, and ethyl acetate on a logarithmic concentration scale are plotted in Figure 8b, Figure 8d and Figure 8f, respectively. In all cases, the response magnitude monotonically increased with increasing analyte concentration, demonstrating a clear dose-dependent relationship. These results confirm that the ZIF-8-coated Au nano-urchin substrate can sensitively detect multiple classes of VOCs, with a reproducible and concentration-dependent optical response. The limits of detection (LODs) were determined using a regression-based approach. The calibration data of ∆A versus analyte concentration were linearly fitted within the low-concentration range, and the residual standard error (δ) was obtained from the fit. The LOD was then calculated as follows:

(5) $$LOD=3.3\frac{\delta }{S},$$

where S is the slope of the fitted calibration curve representing the sensitivity. This regression-based method was applied consistently to 2-pentanone, acetic acid, and ethyl acetate, yielding LOD values of 12.7 ppm, 14.5 ppm, and 36.3 ppm, respectively.

**Figure 9:**
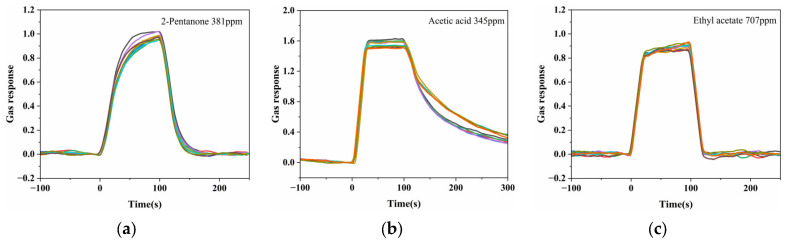
Repeatability of LSPR responses of the ZIF-8–modified Au nano-urchin substrate to three representative VOC vapors: (**a**) 381 ppm 2-pentanone, (**b**) 345 ppm acetic acid, and (**c**) 707 ppm ethyl acetate. Each color line represents one of the ten consecutive exposure–purge cycles.

The repeatability of the ZIF-8–modified Au nano-urchin substrate was evaluated by ten consecutive exposure–purge cycles for three representative VOCs: 381 ppm 2-pentanone, 345 ppm acetic acid, and 707 ppm ethyl acetate. As shown in Figure 9, the baseline-corrected LSPR responses were highly consistent across all cycles, with RSD values of 3.96%, 2.64%, and 2.51%, respectively. These results demonstrate excellent short-term repeatability and suggest good structural stability of the plasmonic substrate during repeated operation. No noticeable degradation in optical or sensing performance was observed during the entire experimental period, indicating the potential durability of the substrate for extended use.

**Figure 10:**
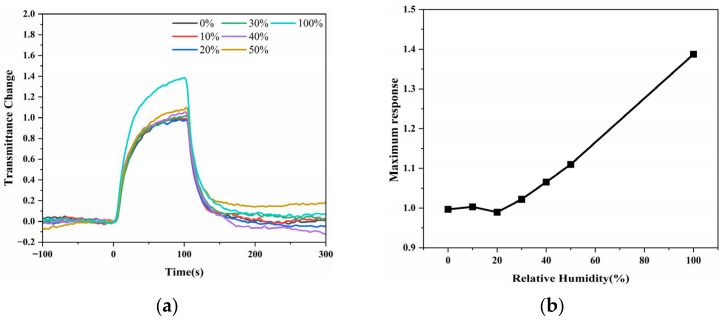
Humidity-dependent LSPR response of the ZIF-8–modified Au nano-urchin substrate using 2-pentanone as a representative analyte. (**a**) Real-time transmission intensity changes at 600 nm during gas exposure (0–100 s) and air purging under different relative-humidity (RH) levels (0%, 10%, 20%, 30%, 40%, 50%, and 100%). (**b**) Maximum response amplitude as a function of RH.

The effect of ambient humidity on the sensor response was evaluated using 2-pentanone as a representative analyte (Figure 10a,b). Measurements were conducted under controlled relative-humidity levels of 0%, 10%, 20%, 30%, 40%, 50%, and 100%. The LSPR signal at 600 nm showed rapid and reversible variations during gas exposure (0–100 s) and purging for all humidity levels. As summarized in Figure 10b, the response magnitude changed only slightly below 20% RH, whereas a linear dependence (y = 0.53x + 0.8552) was observed above this range. These results indicate that the ZIF-8–modified substrate exhibits good humidity tolerance and a predictable linear behavior under higher humidity conditions. At normal ambient temperatures, the refractive-index variation in air and the corresponding shift in the localized surface plasmon resonance spectrum are far smaller than the gas-induced signal magnitude, indicating that temperature fluctuations have a negligible influence on the sensing performance under typical laboratory conditions.

### 3.3. Visualization of Gas Distributions Using the Camera-Based LSPR System

To further demonstrate the capability of the ZIF-8-modified Au nano-urchin substrate for spatial gas sensing, a camera-based LSPR visualization platform was used. In this configuration, the target VOCs generated by the gas generation system were directed onto the substrate surface through a PTFE tube, and the transmitted light images were recorded by a FLIR Blackfly S camera. Each experiment consisted of a sequence of 60 s clean air exposure, 120 s target-gas exposure, and 120 s purging with air. The raw images were processed by frame-to-frame subtraction, noise reduction, and pseudo-color mapping, resulting in differential intensity heatmaps that clearly revealed the gas plume evolution.

**Figure 11:**
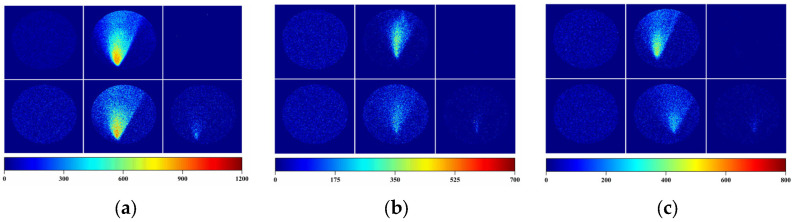
Visualization of gas plumes using the camera-based LSPR system with a ZIF-8-modified Au nano-urchin substrate. (**a**) Heatmaps of 2-pentanone plumes. The first row is for 381 ppm 2-pentanone (20 mL vial, 0.5 L/min), and second row is for 149 ppm 2-pentanone (20 mL vial, 1.0 L/min). The columns correspond to the baseline (0 s, left panel), after 60 s of gas exposure (middle panel), and after ~60 s of air purging (right panel). (**b**) Heatmaps of acetic acid plumes. The first row is for 35 ppm acetic acid (D30 tube, 0.5 L/min), and the second row is for 17 ppm acetic acid (D30 tube, 1.0 L/min). (**c**) Heatmaps of ethyl acetate plumes. The first row is for 131 ppm ethyl acetate (20 mL vial, 0.5 L/min), and the second row is for 65 ppm ethyl acetate (20 mL vial, 1.0 L/min). Higher analyte concentrations consistently produced brighter and more extended conical response regions than lower analyte concentrations.

Visualization of 381 ppm 2-pentanone generated from a 20 mL glass vial at a 0.5 L/min flow rate is shown in the first row of Figure 11a. The initial differential image showed a uniform baseline (left panel). After 60 s of gas exposure, a bright conical region appeared (middle panel), with the highest intensity at the nozzle impingement area and a gradual decrease in the intensity with the distance from the nozzle, clearly visualizing the upward-directed gas plume. Upon switching back to clean air for ~60 s, the response largely recovered to the pre-exposure state (right panel). The second row in Figure 11a shows the response to 149 ppm 2-pentanone at a 1.0 L/min flow rate. Compared with the findings with the higher 2-pentanone concentration, the plume area and intensity were smaller, and the signal decreased more rapidly with the distance from the nozzle, which is consistent with the lower analyte concentration. The results of analogous experiments for acetic acid are shown in Figure 11b. The first row corresponds to 35 ppm acetic acid generated at 0.5 L/min using a D30 tube, while the second row corresponds to 17 ppm acetic acid generated at 1.0 L/min. In both cases, the conical gas plume was clearly resolved, but the higher acetic acid concentration (35 ppm) produced a larger and brighter response region than the lower acetic acid concentration (17 ppm). The results for ethyl acetate are shown in Figure 11c. The first row shows the results for 131 ppm ethyl acetate generated at 0.5 L/min, and the second row shows the results for 65 ppm ethyl acetate generated at 1.0 L/min. The same trend was observed: the higher ethyl acetate concentration produced a stronger and more extended response region, whereas the lower ethyl acetate concentration produced smaller, weaker plumes. Notably, in the experiments performed at the higher flow rate of 1.0 L/min (second rows of Figure 11a–c), a weak residual signal was observed in the gas-impingement region even after 60 s of air purging. This residual response can be attributed to the slight surface damage or perturbation of the substrate induced by the higher flow rate. Nevertheless, the effect was minor and did not significantly affect the overall sensing or visualization performance.

Overall, these differential heatmaps demonstrate that the camera-based LSPR detection system, combined with the ZIF-8-modified Au nano-urchin substrate, enables direct visualization of gas distributions. Moreover, the method exhibits a clear dependence on the analyte concentration, allowing not only the detection of the presence of VOCs, but also approximate discrimination of the relative concentration levels.

### 3.4. Visualization of VOC Evaporation Using the Droplet Evaporation Method

In the droplet evaporation experiments, 30 μL of liquid analyte was pipetted into a small aluminum cup placed on the light guide, while the LSPR substrate was mounted face down above the evaporation source. A total of 3600 images were captured over 36 min. The average of the final five frames was used as the baseline, and differential images were generated by subtracting this baseline from each frame.

**Figure 12:**
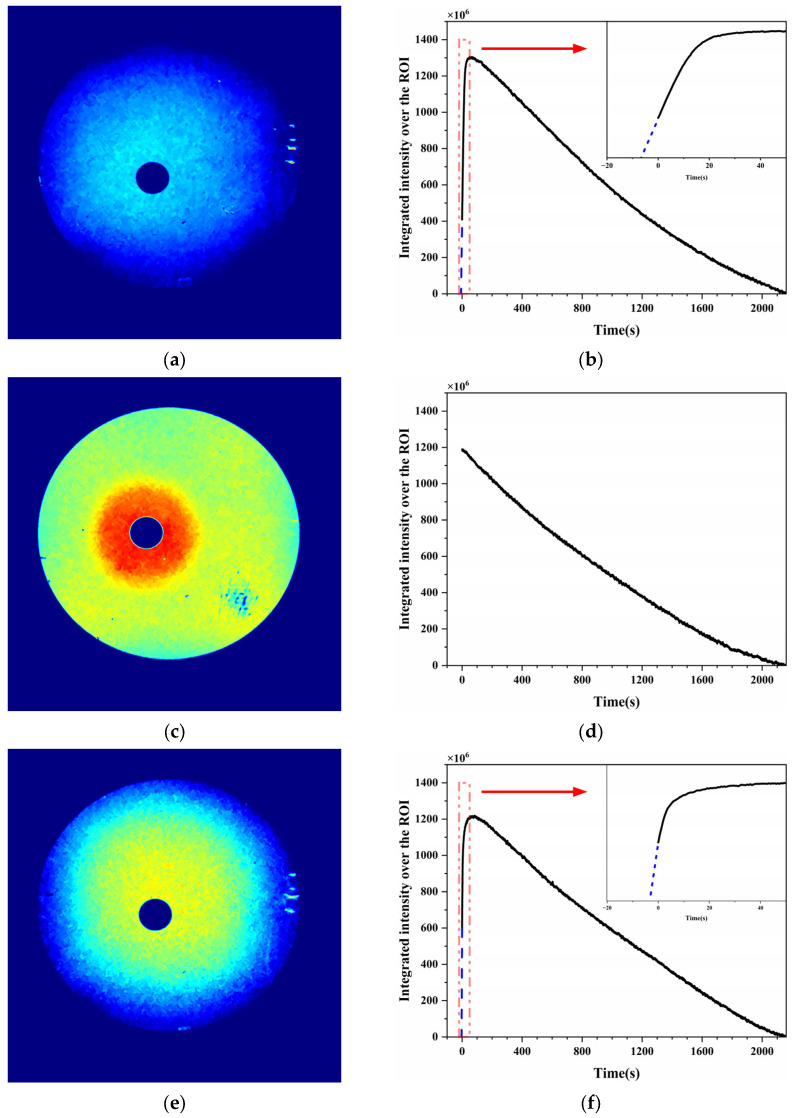
Visualization of VOC plumes generated by natural evaporation of 30 μL liquid droplets in an aluminum cup with the LSPR substrate mounted face down. 2-Pentanone: (**a**) differential heatmap showing a vapor plume centered at the source and (**b**) integrated response curve, with a rapid initial increase (the insert shows the linear increase, with the blue dashed line extrapolated from the linear region) followed by a gradual decrease in the intensity. Aqueous acetic acid (1%): (**c**) initial heatmap showing the maximum plume intensity and (**d**) the integrated response monotonically decreasing with time owing to the high initial concentration. Ethyl acetate: (**e**) plume distribution similar to that of 2-pentanone and (**f**) integrated response showing a rapid increase and then a subsequent decrease in the intensity. In (**a**,**c**,**e**), pseudo-color maps (cv2.COLORMAP_JET) are used, where red represents stronger transmittance changes and blue indicates weaker changes. In (**b**,**f**), the blue dashed lines indicate the linear fitting of the early-stage growth region.

For 2-pentanone (Figure 12a,b), the initial differential heatmap (Figure 12a) showed a circular high-response region centered above the evaporation source, confirming upward diffusion of the vapor. The integrated intensity over the region of interest (ROI) rapidly increased at the beginning and reached a peak within the first few minutes. The early-stage response, which exhibited an approximately linear increase, is shown in Figure 12b’s insert. The dashed blue line was extrapolated from the linearly increasing region to estimate the plume growth prior to camera triggering. After reaching the maximum, the signal gradually decreased as the evaporation rate decreased.

For acetic acid (Figure 12c,d), a 1% aqueous solution was used to avoid the substrate damage caused by long-term exposure to highly concentrated vapor. The initial differential heatmap (Figure 12c) showed a strong central response, indicating that the plume had already reached its maximum at the start of recording. The integrated intensity (Figure 12d) then monotonically decreased with time, suggesting that most of the vapor release occurred before image acquisition began.

For ethyl acetate (Figure 12e,f), the differential image again showed a circular plume centered at the evaporation source. The temporal response curve (Figure 12f) resembled that of 2-pentanone: a sharp increase during the early stage followed by a slow decrease. The intensity was extrapolated to approximate the pre-recording response (insert of Figure 12f), confirming a nearly linear initial increase.

The distinct plume evolution behaviors observed for 2-pentanone, acetic acid, and ethyl acetate can be attributed to their physicochemical properties, including vapor pressure, boiling point, and intermolecular interactions. Acetic acid, which exhibits strong hydrogen bonding and low volatility, evaporates slowly and tends to locally accumulate near the evaporation source. This results in much higher signal intensity close to the source and a longer persistence of the high-intensity region over time. In contrast, the more volatile 2-pentanone and ethyl acetate diffuse rapidly, producing more spatially uniform intensity distributions across the substrate and shorter durations of elevated signal intensity. These trends are consistent with the known vaporization and diffusion characteristics of these compounds.

The results confirmed that the camera-based LSPR system can capture the spatiotemporal evolution of VOC plumes generated by natural evaporation, and distinct behaviors were observed depending on the analyte volatility and concentration.

## 4. Conclusions

We developed a ZIF-8–modified Au nano-urchin LSPR substrate that enables both sensitive detection and visualization of VOCs. The substrate prepared with the optimized ZIF-8 growth time of 90 min exhibited low detection limits of 12.7, 14.5, and 36.3 ppm for 2-pentanone, acetic acid, and ethyl acetate, respectively. Importantly, integration of the substrate with a camera-based LSPR platform allowed real-time visualization of gas plumes under both controlled flow and natural evaporation conditions, providing intuitive spatial information beyond that obtained from conventional point sensors.

This work demonstrates the promise of MOF–plasmonic substrates not only for sensitive and reproducible VOC sensing but also for potential deployment in environmental monitoring, industrial safety, and healthcare diagnostics. The fabrication route—solution-phase MOF growth on APTES-treated glass combined with colloidal Au nano-urchin deposition—is in principle scalable to large areas; however, maintaining film uniformity and reproducible plasmonic coupling across batches remains a key challenge.

Future work will focus on improving fabrication control and exploring cost-effective patterning or printing methods for large-scale production. In addition, introducing functional coatings or tailoring surface terminal groups (e.g., polymer or thiol-based layers, MOF composites) could impart enhanced molecular selectivity. Furthermore, coupling this platform with SERS readouts will allow simultaneous wide-field visualization and molecular fingerprint identification. These advances are expected to improve both selectivity and scalability, paving the way for field-deployable, multimodal plasmonic sensing systems.

## Data Availability

The data are contained within the article and Supplementary Materials.

## Supplementary Materials

The following supporting information can be downloaded at https://doi.org/10.5281/zenodo.17308968 (accessed on 20 October 2025). Figure S1: Scanning electron microscopy images of the Au nano-urchin substrates produced with different ZIF-8 growth times; Video S1: Visualization of the 2-pentanone vapor distribution during the droplet evaporation experiment; Video S2: Visualization of the 2-pentanone vapor distribution at 149 ppm 2-pentanone using the gas delivery system; Video S3: Visualization of the acetic acid vapor distribution at 35 ppm acetic acid using the gas delivery system; Video S4: Visualization of the acetic acid vapor distribution at 17 ppm acetic acid using the gas delivery system; Video S5: Visualization of the ethyl acetate vapor distribution at 131 ppm ethyl acetate using the gas delivery system; Video S6: Visualization of the ethyl acetate vapor distribution at 65 ppm ethyl acetate using the gas delivery system; Video S7: Visualization of the 2-pentanone vapor distribution during the droplet evaporation experiment; Video S8: Visualization of the acetic acid vapor distribution during the droplet evaporation experiment; Video S9: Visualization of the ethyl acetate vapor distribution during the droplet evaporation experiment.
